# Estimating the effect of antenatal care models on birthweight in low- and middle-income countries: Evidence from a DHS-based propensity score matching analysis

**DOI:** 10.1371/journal.pone.0355685

**Published:** 2026-08-14

**Authors:** Aklilu Habte Hailegebireal, Demelash Woldeyohannes Handiso

**Affiliations:** 1 School of Public Health, College of Medicine and Health Sciences, Wachemo University, Hosanna, Ethiopia; 2 Faculty of Health and Environmental Sciences, School of Acute and Primary Health, Auckland University of Technology, Auckland, New Zealand; 3 Monash Centre for Health Research and Implementation, Monash University, Melbourne, Australia; LMU München: Ludwig-Maximilians-Universitat Munchen, NEPAL

## Abstract

Low birthweight (LBW) remains a major public health challenge in low- and middle-income countries (LMICs), accounting for over 95% of the global burden and substantially increasing the risks of neonatal mortality, impaired growth, and adverse lifelong health outcomes. Despite the recognized importance of antenatal care (ANC), robust evidence on the causal effect of different ANC models on birthweight across LMICs remains limited. This study aimed to estimate the effect of adequate ANC utilization on birthweight using propensity score matching (PSM).

**Method:**

This study analysed 370,576 women (weighted sample = 369,034) drawn from 52 Demographic and Health Surveys(DHS) conducted across LMICs between 2012 and 2024. Potential confounders associated with both ANC utilization and birthweight were identified using bivariate analyses, including chi-square tests, analysis of variance (ANOVA), and t-tests, and were subsequently incorporated into PSM models. PSM was employed to estimate the effects of receiving at least four ANC visits (ANC4+) and at least eight ANC visits (ANC8+) on birthweight. Both the Average Treatment Effect on the Treated (ATT) and the Average Treatment Effect (ATE) were estimated. Matching quality was assessed using balance diagnostics and graphical evaluations, while Rosenbaum bounds sensitivity analysis was performed to evaluate the robustness of the findings to potential unmeasured confounding.

**Results:**

The mean birthweight was 3,034.31 g (SD: 651.65), and the weighted prevalence of LBW was 12.42% (95% CI: 12.31, 12.52). Overall, 69.95% and 20.73% of women received at least four (ANC4+) and eight (ANC8+) ANC visits, respectively. PSM revealed that women who received ANC4+ and ANC8+ delivered newborns weighing, on average, 76.41 g and 104.16 g more than matched controls, respectively (ATT). At the population level, ANC4+ and ANC8+ were associated with average birthweight gains of 60.18 g and 84.61 g, respectively (ATE). Matching diagnostics demonstrated substantial reductions in covariate imbalance and strong post-matching comparability between treatment and control groups, supporting the robustness of the estimated treatment effects.

**Conclusion:**

Low birthweight remains a substantial public health burden in LMICs. Adequate ANC was associated with significant improvements in birthweight, with the WHO-recommended ANC8+ model demonstrating greater benefits than ANC4+, indicating a dose-dependent relationship between ANC utilization and fetal growth. Expanding access to and continuity of high-quality ANC services, particularly the ANC8+ contact model, may represent an effective strategy for improving birth outcomes and reducing the burden of LBW across the region.

## Background

Birth weight refers to the first recorded weight of a newborn, ideally measured within the initial hours following delivery [1]. According to the World Health Organization (WHO), a newborn is classified as having low birth weight (LBW) if the weight is below 2,500 grams [2,3]. It reflects complex public health issues, such as maternal malnutrition and inadequate prenatal care, and is recognized as a key global health indicator by both the WHO and the Global Nutrition Monitoring Framework [2].

Globally, approximately 19.8 million infants, representing one in seven newborns (14.7% of all live births), are born with low birthweight (LBW) [3,4]. An overwhelming majority of LBW cases (95.6%) occur in low- and middle-income countries (LMICs). Of these, nearly two-thirds were born in Asia, with Southern Asia alone contributing over 40% of the global LBW burden, while Africa accounts for approximately one-third of LBW births, predominantly concentrated in Eastern and Western Africa [3,5]. LBW remains a major public health concern because it is strongly associated with increased risks of neonatal morbidity and mortality, impaired immune function, growth restriction, poor cognitive development, reduced educational attainment, and adverse health outcomes later in life, including non-communicable diseases [3,6–9]. Infants born with LBW face a mortality risk approximately 20 times higher than their heavier counterparts [2,3].

The disproportionate burden of LBW in LMICs reflects persistent challenges such as chronic maternal undernutrition [10,11], infections during gestation such as malaria [12–14], polyparasitism [15,16], and COVID-19 [17,18], as well as maternal anaemia [19,20], hypertensive disorders [21–23], and multiple pregnancies [24,25]. Additionally, behavioural exposures, particularly tobacco use [26–28], alcohol consumption, and substance use [29–31] during gestation, further elevate the likelihood of LBW outcomes.

Timely and adequate antenatal care (ANC) offers a critical opportunity to prevent, detect, and manage many of the modifiable risk factors associated with low birth weight (LBW) [5,32–34]. By facilitating access to essential maternal health services, ANC helps mitigate conditions such as malnutrition, untreated illnesses, and gaps in health education. Its strategic role is central to achieving the World Health Assembly (WHA) endorsed comprehensive implementation plan on maternal, infant, and young child nutrition goal of reducing LBW prevalence by 30% between 2012 and 2030 [3,35]. The WHO’s former Focused (four-visit) ANC model (ANC4+) was updated in 2016 to recommend a minimum of eight contacts (ANC8+) [36]. Expanding ANC coverage and quality is vital, especially in LMICs known for a high burden of LBW, for improving birth outcomes [37].

Low birth weight remains a pressing public health challenge in low- and middle-income countries, where disparities in maternal healthcare access drive its persistently high burden [38,39]. Although the burden of LBW in LMICs has been well studied, evidence on the effectiveness of adequate ANC in reducing LBW remains limited. In particular, few studies have explored the comparative impact of receiving four or more ANC visits (ANC4+) versus the WHO-recommended eight or more contacts (ANC8+), leaving a critical gap in understanding how this policy shift translates into improved birth outcomes.

Although several studies have documented the burden of LBW in LMICs [25,37,38,40], there remains a notable lack of evidence on the impact of maternal healthcare interventions, particularly ANC, on LBW. In particular, limited evidence exists on the comparative impact of receiving four or more antenatal care visits (ANC4+) versus the WHO-recommended eight or more contacts (ANC8+), creating a gap in understanding how this shift influences tangible health outcomes like reductions in low birth weight. While some studies have reported associations between ANC quality and birth weight [41–44], little is known with robust methods like propensity score matching (PSM) to estimate the actual causal effect.

This study seeks to fill an evidence gap by examining how adequate ANC influences birth weight outcomes in LMICs. To strengthen causal inference, the analysis employs propensity score matching (PSM), minimizing confounding and selection bias inherent in observational data [45]. By comparing the effects of the traditional four-visit ANC model (ANC4+) with the updated eight-contact standard (ANC8+), the study offers valuable insights for refining policy frameworks, optimizing service delivery, and informing strategic resource allocation. These findings have the potential to accelerate progress toward Sustainable Development Goal 3 (SDG 3) and Global Nutrition Target of a 30% reduction in LBW—an objective now extended to 2030 in response to slower-than-anticipated gains.

## Methods

### Study design, period, and population

This study employed a cross-sectional design using pooled, publicly available data from the Demographic and Health Surveys (DHS) conducted in LMICs. The study population comprised women aged 15–49 years who had a live birth within the five years preceding each survey, consistent with the DHS birth recode file. The most recent DHS surveys conducted between 2012 and 2024 were considered for inclusion. To ensure comparability across countries, only surveys containing complete information on birthweight, ANC utilization, and the covariates included in the propensity score matching analysis were retained. Records with missing birthweight, incomplete ANC information, or multiple births were excluded. The final pooled dataset comprised a weighted sample of 369,034 eligible women from 52 LMICs (Table 1).

**Table 1:** Description of the countries included with their respective unweighted and weighted sample size for ANC4+, ANC8+, and LBW 2012-2024.

Region	Country	Year	Unweighted sample	Weighed Sample Size (N)	ANC4+(n)	ANC8+(n)	LBW(n)
Central Asia	Kyrgyz Republic	2012	3,052	2,918	2444	913	139
	Tajikistan	2023	3,780	3,927	2626	598	233
	Total		6,832	6,845	5071	1511	372
Latin America and the Caribbean	Colombia	2015	7,850	7,773	7160	3521	659
	Dominica Republic	2013	2,791	2,773	2661	1905	368
	Guatemala	2015	8,791	8,854	7706	3498	1,173
	Honduras	2012	6,971	6,882	6285	2649	569
	Haiti	2017	1,578	1,663	1386	360	275
	Peru	2012	7,313	6,823	6536	4603	404
	Total		35,294	34,768	31734	16536	3,448
Sub-Saharan Africa	Angola	2016	4,677	4,914	3873	501	431
	Burkina Faso	2021	5,160	5,130	3824	44	512
	Benin	2018	5,343	5,345	3293	579	501
	Burundi	2021	6,773	6,923	3562	34	600
	Congo Democratic Republic	2024	7,341	7,846	4250	213	457
	Congo Brazzaville	2012	5,175	5,098	4215	277	421
	Cote d’Ivoire	2021	4,007	3,906	2522	187	459
	Cameroon	2018	4154	4,162	3369	457	230
	Ethiopia	2016	1,657	1,216	828	97	139
	Gabon	2021	3,699	3,788	3148	519	455
	Ghana	2022	3,873	3,601	3329	1536	306
	Gambia	2020	4,248	4,144	3294	186	323
	Guinea	2021	2,478	2,561	1271	121	227
	Kenya	2022	4,084	4,088	2878	171	291
	Comoros	2012	1,207	1,242	761	172	201
	Liberia	2022	1,375	1,284	1181	385	108
	Lesotho	2024	2,069	2,069	1622	288	193
	Madagascar	2021	3,582	3,688	2913	146	428
	Mali	2018	2,278	2,402	1487	130	315
	Mauritania	2021	1,399	1,340	708	80	320
	Malawi	2016	11,135	11,066	5785	156	1,185
	Mozambique	2023	3,149	3,003	1935	85	348
	Nigeria	2021	4,972	5,255	4671	2419	343
	Niger	2012	2,461	2,038	937	5	200
	Namibia	2013	2620	2,510	2113	733	283
	Rwanda	2020	5,652	5,778	2829	16	307
	Sierra Leone	2019	4279	4,209	3837	1016	194
	Senegal	2023	3,709	3,544	2742	301	516
	Chad	2015	1214	1,386	855	24	91
	Togo	2014	2,906	2,897	1980	121	238
	Tanzania	2022	4,381	4,447	3113	159	303
	Uganda	2016	6,788	6,733	4404	134	530
	South Africa	2016	2,555	2,569	2034	471	351
	Zambia	2018	5,730	5,818	3851	79	411
	Zimbabwe	2015	4,055	4,054	3284	552	308
	**Total**		**140,185**	**140,054**	**96695**	**12391**	**12,523**
South and southeast Asia	Afghanistan	2015	2,601	2,863	1215	184	436
	Bangladesh	2022	3,110	3,150	1584	224	429
	India	2021	154,976	154,614	95911	32280	26,142
	Indonesia	2017	13,907	13,987	13001	9369	840
	Myanmar	2016	1745	1,698	1334	490	125
	Maldives	2017	2,171	1,919	1860	1608	225
	Nepal	2022	4,452	4,345	3765	313	483
	Philippines	2022	3,764	3,647	3223	1235	562
	Pakistan	2018	1542	1,143	895	343	256
	Total		**188,268**	**187,367**	**122788**	**46046**	**29,498**
**Total**	**52**	**2012-2024**	**370,576**	**369,034**	**256288**	**76483**	**45,841**

### Sampling techniques and procedures

**Fig 1:**
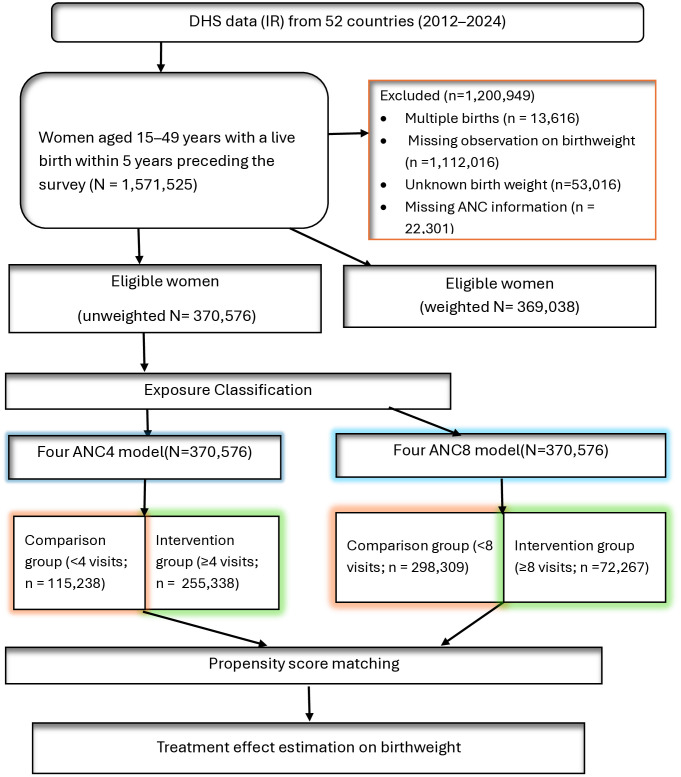
Flow diagram illustrating participant selection, eligibility assessment, exposure classification, and propensity score matching analyses.

The DHS employed a two-stage stratified cluster sampling design to ensure national representativeness across low- and middle-income countries. In the first stage, clusters, defined as enumeration areas(EAs), were selected using probability proportional to size sampling. In the second stage, a fixed number of households within each cluster were systematically sampled. The details are mentioned elsewhere [46,47]. This study utilized pooled Individual Recode (IR) data from DHS conducted in 52 LMICs. A total of 1,571,525 women aged 15–49 years who had a live birth within the five years preceding the survey were initially identified. Records from multiple births, those with missing birthweight information, unknown birthweight, and missing ANC information were excluded. Finally, 370,576 women remained eligible for analysis. After applying DHS sampling weights, the corresponding weighted analytical sample comprised 369,034 women. The eligible women were subsequently classified according to two ANC exposure definitions based on World Health Organization recommendations. For the ANC4 model, women were categorized into a comparison group (<4 ANC visits) and an intervention group (≥4 ANC visits). For the ANC8 model, women were categorized into a comparison group (<8 ANC visits) and an intervention group (≥8 ANC visits). Propensity score matching was then performed separately for the ANC4 and ANC8 models to estimate the effect of ANC utilization on birthweight while minimizing observed differences between the comparison and intervention groups (Fig 1).

### Measurement of variables of the study

#### Outcome variable:.

The primary outcome variable is birthweight, defined as the first recorded weight of the newborn immediately after delivery. It was measured as a continuous variable in grams and obtained from either documented health card/medical records or maternal recall, in accordance with standard DHS data collection procedures. DHS interviewers do not directly measure birthweight during survey administration. To improve data quality and comparability across countries, newborns without recorded birthweight measurements and records with missing or implausible values (9998 and 9999) were excluded from the analysis. Modeling birthweight as a continuous outcome enabled a more precise assessment of the effects of antenatal care exposure (ANC4+ and ANC8+) while preserving statistical power and avoiding the loss of information associated with categorizing birthweight into discrete groups [48, 49]. Additionally, birthweight was dichotomized to estimate the weighted prevalence of low birthweight (LBW), with newborns weighing less than 2,500 grams classified as LBW (“Yes” = 1) and those weighing ≥2,500 grams or more classified as normal birthweight (“No” = 0), in accordance with World Health Organization (WHO) recommendations.

### The exposure (treatment) variable

The main exposure variable is antenatal care (ANC), representing the total number of ANC visits a woman received during her most recent pregnancy. This discrete numeric variable captures frequency of ANC contacts, and for analytical purposes, it was dichotomized into two distinct treatment groups as ANC4+ (four or more visits), reflecting former WHO recommendations [50, 51], and ANC8+ (eight or more visits), consistent with current standards [36, 52]. Each group was deemed as a binary variable: women receiving ANC4+ or ANC8+ were assigned a value of 1 = Yes, while those with fewer visits were assigned 0 = No. Two separate models were estimated: Model 1 evaluated the causal effect of ANC4+ on birthweight, and Model 2 assessed the impact of ANC8+. In each model, the control group comprised women who did not meet the respective ANC threshold.

### Explanatory variables

Given the observational nature of the DHS, random assignment of exposure was not applied, leading to inherent differences between treatment and control groups. To address potential selection bias in estimating the effects of ANC4+ and ANC8+ on birthweight, we identified maternal baseline characteristics that influence both the exposures(ANC) and the outcome (birth weight) based on prior literature [25, 52].

A range of sociodemographic, reproductive, and health-related factors was included as independent variables. Sociodemographic characteristics included age, marital status, education, residence, family size, sex of the household head, media exposure, and household wealth. Age was recorded in completed years and categorized into 15–29, 20–34, and 35–49 years; Marital status (married, never married, or formerly married (including divorced, widowed, and separated); and women’s level of education (no education, primary, secondary, or higher education). The place of residence was defined based on administrative classification and grouped as urban or rural. Family size represented the total number of individuals residing in the household and was grouped as fewer than five (<5) or five or above (≥5). The sex of the household head was recorded as male or female. Media exposure was assessed based on the frequency with which women engaged with newspapers, television, or radio, and categorised as not at all, less than once a week, or at least once a week. Economic status was measured using a wealth index derived from principal components analysis (PCA) of household assets and living conditions, and households were ranked into five quintiles: poorest, poorer, middle, richer, and richest.

Reproductive and maternal health variables included parity, preceding birth interval, pregnancy intention, contraceptive use, timing of first antenatal care (ANC) visit, and iron supplementation. Parity was defined as the number of living children and categorized as nulliparous (0), primiparous (1), multiparous (2–4), and grand multiparous (≥5). The preceding birth interval measured the number of months between the birth of the index child and the immediately preceding sibling, categorized as first birth (no interval), less than 12 months, 12–23 months, and 24 months or more. Pregnancy intention was self-reported and categorized as wanted then (planned), wanted later (mistimed), or not wanted at all (unwanted). Contraceptive uptake was categorized into non-users, users of hormonal methods (injectables, implants, pills), and users of non-hormonal methods (including intrauterine devices, condoms, diaphragm, permanent methods such as tubal ligation or vasectomy, lactational amenorrhea method, standard days method, and traditional methods). Timing of the first ANC visit was recorded in months of gestation and categorized as early (first trimester, ≤3 months) or late (second or third trimester, >3 months). Iron intake was assessed based on self-reported consumption of iron-containing supplements during the most recent pregnancy and categorized as yes or no.

Behavioural and healthcare access variables included cigarette smoking and barriers to accessing healthcare. Smoking status was determined by self-report and categorized as smoker (yes) or non-smoker (no). Barriers to accessing healthcare were measured through self-reported challenges such as obtaining permission to seek care, securing money for treatment, distance to health facilities, and reluctance to go alone. Each barrier was categorized as either a “big problem” or “not a big problem. Autonomy in decision-making was measured by whether the woman reported having the final say in decisions regarding her own healthcare and was categorized as autonomous (yes) or not autonomous (no).

### Confounding variables and causal framework

**Fig 2:**
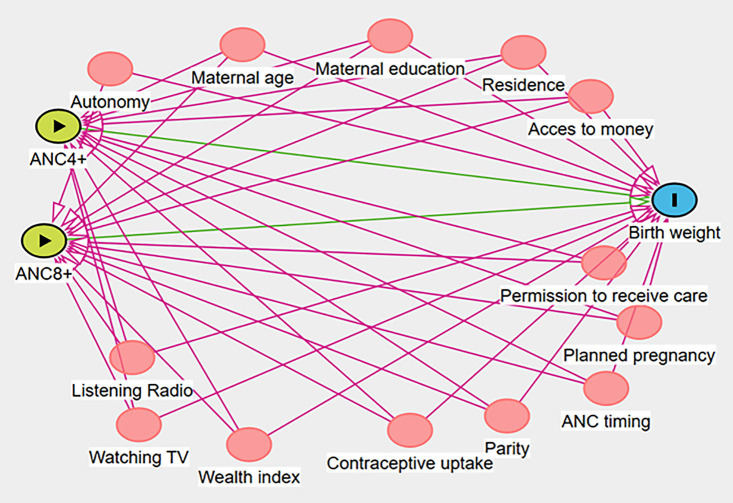
A directed acyclic diagram that shows the relationship between exposure, outcome, and confounders in LMICs.

Confounders were identified through a two-step process combining empirical association and causal reasoning. First, bivariate analyses were conducted to assess the relationship between candidate covariates and both the exposure (ANC4+ and ANC8+) and the outcome (birthweight). Variables that demonstrated statistically significant associations with both were considered potential confounders. Accordingly, the following covariates were identified: maternal age, level of education, place of residence (urban/rural), wealth index, parity, ANC timing (early/late), contraceptive uptake, planning status of pregnancy, financial accessibility of care (ease of obtaining money), autonomy in care-seeking (permission to attend a facility), exposure to mass media (radio, television, newspaper), and enrolmnt in health insurance scheme. Then, a Directed Acyclic Graph (DAG) was developed using DAGitty version 3.0 to visualize and validate the hypothesized causal relationships among exposures, outcomes, and covariates (Fig 2).

### Statistical analysis

All data management and statistical analyses were performed using STATA version 18. To account for the complex survey design and ensure nationally representative estimates, analyses were weighted using the sampling weights provided by the DHS Program. Chi-square tests were applied to assess relationships between the exposure variables and categorical covariates. To evaluate differences in mean birthweight across categories of potential covariates, one-way analysis of variance (ANOVA) and independent t-tests were employed, as appropriate. Covariates demonstrating statistically significant associations with both the exposure and the outcome variables were classified as potential confounders and subsequently included in the PSM model to adjust for selection bias and improve causal inference.

### Propensity score matching

This study utilized data from the Demographic and Health Surveys (DHS), a large-scale observational dataset in which exposure groups—specifically ANC4+ and ANC8+—were not randomly assigned [53,54]. As a result, baseline differences between comparison groups may exist, introducing potential confounding and selection bias in estimating the causal effect of antenatal care adequacy on birthweight. To address these imbalances, PSM was employed to construct comparable groups of treated and untreated individuals, thereby enhancing the internal validity of causal inference [45,55]. Separate logit models were fitted for each exposure group to estimate propensity scores, defined as the conditional probability of receiving ANC4+ or ANC8+ given a set of observed confounders [45,56]. These scores, ranging from 0 to 1, were generated using the *pscore* command in STATA and served as the basis for matching individuals across treatment conditions.

Following the creation of matched samples, the effects of ANC4+ and ANC8+ exposure were independently evaluated by comparing mean birthweight (in grams) between treated and control groups. PSM was implemented using logistic regression models via the psmatch2 command in STATA, with maternal exposure to ANC4+ and ANC8+ specified as binary treatment indicators (Yes = 1, No = 0). This approach enabled estimation of average treatment effects within covariate-balanced groups. To assess the adequacy of matching, covariate balance was examined before and after matching using the *pstest* command. A p-value less than 0.05 was interpreted as evidence of residual imbalance, whereas a p-value greater than 0.05 post-matching indicated satisfactory balance between treatment and control groups, supporting the validity of the matched comparisons [57–59].

This study aims to estimate the average effect of receiving eight or more antenatal care visits (ANC8+) on birthweight among mothers who actually received the intervention. Let Y_i_T represent the observed birthweight for the i^th^ newborn whose mother received the treatments (ANC4+ or ANC8+), and Y_i_C denote the counterfactual birthweight among newborns of mothers without the respective treatments. The treatment indicator T is defined as a binary variable, where T = 1 indicates having an exposure and T = 0 denotes non-exposure. This framework allows for the estimation of the average treatment effect on the treated (ATT), capturing the causal impact of each treatment on birthweight within the exposed population. The treatment assignment indicator T equals 1 if the mother received treatment and 0 otherwise. The observed outcome can thus be described as: Y_i_ = T_i_ Y_i1_ + (1 – T_i_) Y_i0_ [60,61] Where

*Y*_*i*_ *: observed birth weight for individual i,*

*T*_*i*_ *: Exposure indicator (1 if exposed to treatment (ANC4+ or ANC8+, 0 otherwise),*

*Y*_*i1*_ *: potential outcome if treated (exposed), which means birth weight if the mother received each treatment*

*Y*_*i0*_ *: potential outcome if not treated (unexposed)*

### Matching strategy and evaluation of PSM assumptions

To mitigate post-treatment bias, covariates were selected before exposure assignment, ensuring that only pre-treatment variables informed the propensity score model. Key assumptions of propensity score matching, namely, common support and unconfoundedness (i.e., selection on observables), were assessed through both graphical diagnostics and statistical tests. Matching was restricted to treated groups whose estimated propensity scores fell within the range observed among control participants, thereby satisfying the common support condition. Multiple matching algorithms were explored, including nearest neighbour matching (with and without replacement) and radius matching using a caliber width of 0.01. Covariate balance before and after matching was evaluated using the pstest command in STATA, with p-values greater than 0.05 interpreted as evidence of adequate balance. Treatment effects—including the average treatment effect on the treated (ATT), untreated (ATU), and overall population (ATE)—were estimated using the *psmatch2* procedure. The final matching specification was selected based on optimal covariate balance and model performance.

Matching quality was assessed using standardized bias metrics, model significance tests, and evaluation of the common support assumption. Visual inspection of post-matching covariate balance was conducted using balance plots. To test the robustness of treatment effects against potential unobserved confounding, Rosenbaum bounds sensitivity analysis was applied for continuous outcomes [62]. The sensitivity parameter Gamma (Γ) quantified the extent to which hidden bias could alter treatment assignment; values of Γ > 1 indicate increasing susceptibility to unmeasured confounding [62–64]. The point at which the treatment effect loses statistical significance offers insight into the credibility of causal inference under non-random assignment.

### Ethical consideration and consent to participate

This study employed publicly accessible, de-identified data from the Demographic and Health Surveys (DHS), which are collected under rigorous ethical protocols, including informed consent from participants and approval by national ethics committees in each participating country. As the dataset is fully anonymized and contains no personally identifiable information, neither ethical approval nor additional informed consent was required for this secondary analysis. The research involved no direct interaction with human subjects and posed no risk to individual privacy or confidentiality. All analyses were conducted in compliance with the DHS data use agreement and aligned with the ethical principles outlined in the Declaration of Helsinki and institutional standards for research involving human data.

## Results

### Baseline characteristics of the study population

This analysis is based on a weighted sample of 369,034 women in 52 low and middle-income countries, with the largest and lowest contributions from South & Southeast Asia (50.8%) and central Asia (1.8%), respectively. The mean age of respondents was 29.26 years (±7.07), and over half (52.0%) were in the age group 25–34 years. The majority (45.7%)of women attended secondary education. A significant portion resides in rural areas (60.8%), and wealth distribution appears relatively balanced across quintiles. Overall, the proportion of women receiving ≥4 ANC visits was notably higher in Latin America & the Caribbean (91.3%) and among those with higher education (80.8%), urban residence (78.2%), and in the richest wealth quintile (77.5%). Similarly, the highest uptake of ANC8+ was seen in Latin America & the Caribbean (47.6%) and among women with higher education (38.1%), while the lowest was among those with no education (7.7%) and in Sub-Saharan Africa (8.9%). The highest mean birthweights were recorded in Central Asia (3281.9g) and among women aged 35–49 (3148.0g), while the lowest were in South & Southeast Asia (2876.7g) and among the poorest (2976.1g). Urban residence, higher education, and wealth were consistently associated with higher birthweight averages (p < 0.005) (Table 2).

**Table 2:** The distribution of sociodemographic characteristics of respondents across adequacy of ANC (ANC4+ and ANC8+) and birth weight in LMICs, 2025.

Variable categories	Total (N=369,034)	≥4 ANC visit		≥8 visits		Birth weight (weighted estimates)	
		Yes(%)	p-value^*^	Yes(%)	p-value^*^	Mean birthweight (g) (95%CI)	p-value^**^
**Region**							
South & Southeast Asia	187,367(50.8)	65.5	<0.001	24.6	<0.001	2876.7(2874.1, 2879.4)	<0.001^***^
Sub-Saharan Africa	140,054 (38.0)	69.4		8.9		3213.1(3209.5, 3216.7)	
Latin America & Caribbean	34,768(9.4)	91.3		47.6		3194.7(3188.2, 3201.2)	
Central Asia	6,8459(1.8)	74.1		22.1		3281.9(3269.0, 3294.7)	
**Age**							
15-24	116,684 (31.6)	67.6	<0.001	17.3	<0.001	2984.6(2980.9, 2988.3)	<0.001^***^
25-34	192,043(52.0)	70.0		22.3		3040.9(3038.0, 3043.7)	
35-49	60,307(16.3)	71.2		22.2		3148.0(3142.8, 3153.2)	
**Educational level**							
No education	64,522(17.5)	54.9	<0.001	7.7	<0.001	2987.6(2982.6, 2992.6)	<0.001^***^
Primary	84,571(22.9)	66.6		13.9		3125.0(3120.5, 3129.7)	
Secondary	168,564(45.7)	73.0		23.8		3018.5(3015.5, 3021.5)	
Higher	51,377(13.9)	80.8		38.1		3049.4(3044.1, 3054.7)	
**Residence**							
Urban	144,489(39.2)	78.2	<0.001^*^	28.1	<0.001	3109.9(3106.4, 3113.6)	<0.001
Rural	224,545(60.8)	63.8		16.0		3005.7(3003.1, 3008.3)	
**Wealth index**							
Poorest	67,473(18.2)	60.5	<0.001^*^	12.4	<0.001	2976.1(2971.6, 2980.6)	0.034^***^
Poorer	72,945(19.8)	65.8		16.7		3018.0(3013.5, 3022.6)	
Middle	74,463(20.2)	69.4		20.8		3049.6(3044.9, 3054.2)	
Richer	77,350(21.0)	72.9		24.1		3068.5(3063.8, 3073.2)	
Richest	76,802(20.8)	77.5		28.4		3109.2(3104.3, 3114.0)	
**Family size**							
≤5 members	181,687(49.2)	72.3	<0.001^*^	24.15	<0.001	3038.0(3035.1, 3041.0)	0.673^**^
>5 members	187,347(50.8)	66.7		17.4		3045.3(3042.3, 3048.2)	
**Head of household**							
Male	297,603(80.6)	69.2	0.011^*^	20.9	<0.001	3032.6(3030.3, 3034.9)	0.064^**^
Female	71,431(19.4)	70.4		20.2		3079.2(3074.4, 3084.1)	

p-value* for chi-square test.

P-value** for an independent t-test.

p-value *** for one-way ANOVA.

### Adequate ANC uptake and birthweight across health service-related characteristics

**Fig 3:**
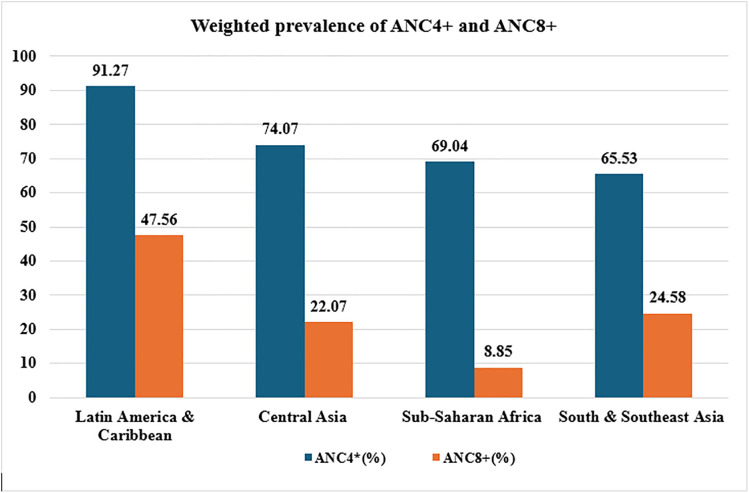
Receipt of adequate Antenatal care in LMICs, 2025.

Overall, 69.45% of women received ANC4+ (95% CI: 69.39, 69.51), while 20.73% received ANC8+ (95% CI: 20.59, 20.89). The highest uptake of both ANC4+ and ANC8+ were recorded in Latin America and Caribbean region (Fig 3).

The majority were multiparous (56.3%), with primiparous women accounting for 33.0%. Early antenatal care (ANC) initiation was reported by 61.6% of women. More than three-fourths (78.8%) of pregnancies were planned, and more than half (53.7%) reported contraceptive usage. Smoking during pregnancy was rare (0.6%), and iron supplementation was high at 87.2%. Barriers to maternity care were notable: 25.1% perceived distance as a major issue, 14.0% faced challenges in obtaining permission, and 30.9% cited financial constraints. Media engagement varied, with 43.1% watching TV, 17.8% listening to the radio, and 9.8% reading newspapers at least once a week, respectively. Sanitation access showed that 68.5% had improved toilet facilities, while 24.6% practiced open defecation. Women who initiated ANC early had the highest coverage for both ≥4 visits (80.7%) and ≥8 visits (29.0%). Contraceptive users (77.5%) and those who watched TV at least once a week (75.1%) also showed higher rates of ≥4 ANC visits. Uptake of ≥8 ANC visits was highest among women who read newspapers at least once a week (30.3%), primiparous (25.0%), contraceptive users (24.9%), and those with improved toilet facilities (24.0%). There was a significant difference in mean birth weight across parity groups, with grand multiparous women exhibiting the highest mean birth weight (3220.2 g), compared to primiparous women (2954.9 g)(Table 3).

**Table 3:** The distribution of health service-related characteristics of respondents across ANC4+, ANC8+, and birth weight in LMICs, 2025.

Variable categories	Total (N=369,034)	≥4 ANC visit		≥8 visits		Birth weight (weighted estimates)	
		Yes(%)	p-value^*^	Yes(%)	p-value^*^	Mean birthweight (g) (95%CI)	p-value^**^
**Parity**							
Primiparous	121,770(33.0)	73.6	<0.001	25.0	0.002	2954.9(2950.1, 2959.7)	<0.001^***^
Multiparous	207,740(56.3)	68.6		20.5		3045.5(3041.8, 3049.2)	
Grand multiparous	39,524(10.7)	61.3				3220.2(3211.0, 3229.3)	
**Timing of ANC**							
Early	227,442(61.6)	80.7	<0.001	29.0	<0.001	3008.5(3005.0, 3011.9)	<0.001^**^
Late	141,592(38.4)	51.3		7.4		3075.9(3071.2, 3080.5)	
**Pregnancy status**							
Wanted then	290,872(78.8)	69.6	0.001	21.4	<0.001	3006.3(3003.2, 3009.5)	<0.001^***^
Wanted later	53,978(14.6)	69.8		17.4		3138.7(3131.3, 3146.1)	
Wanted no more	24,184(6.6)	67.2		20.6		3137.8(3125.8, 3149.8)	
**Contraceptive uptake**							
Non-users	170,763(46.3)	72.9	<0.001	17.1	<0.001	3051.4(3047.1, 3055.6)	<0.001^**^
Users	198,271(53.7)	77.5		23.9		3019.7(3015.9, 3023.4)	
**Smoking cigarette**							
Yes	1,944(0.6)	71.4	0.032^*^	19.1	0.555	3013.8(3010.9, 3016.8)	0.321^**^
No	325,213(99.4)	67.2		18.1		3038.8(2995.8, 3082.0)	
Iron intake							
Yes	321,922(87.2)	71.6	<0.001	21.6	<0.001	3072.0(3063.7, 3080.3)	<0.001^**^
No	47,105(12.8)	54.5		14.5		3028.8(3025.8, 3031.8)	
**Perceived distance**							
Big problem	92,639(25.1)	61.9	<0.001	11.8	<0.001	3033.7(3027.8, 3039.5)	0.131^**^
Not a big problem	276,395(74.9)	71.9		23.7		3034.5(3031.4, 3037.7)	
**Getting permission**							
Big problem	51,780(14.0)	59.9	<0.001	11.5	<0.001	3013.1(3005.7, 3021.9)	<0.001^**^
Not a big problem	317,254(86.0)	71.0		22.2		3037.7(3034.7, 3040.6)	
**Getting money**							
Big problem	114,196(30.9)	64.2	<0.001	11.0	<0.001	3015.2(3012.4, 3019.2)	<0.001^**^
Not a big problem	254,838(69.1)	71.8		25.1		3075.6(3070.5, 3080.7)	
**Autonomy in decision making**							
Non-autonomous	95,685(25.9)	70.8	0.134	16.0	<0.001	2991.2(2988.4, 2994.9)	0.023
Autonomous	273,349(74.1)	70.0		22.4		3056.2(3050.5, 3061.8)	
**Watching TV**							
Not all	155,909(42.2)	64.9	<0.001	16.6	<0.001	3015.0(3010.6, 3019.4)	0.067^***^
< once a week	54,012(14.6)	65.7		16.5		2964.6(2957.8, 2971.4)	
At least once	159,114(43.1)	75.1		26.2		3068.2(3073.9, 3082.4)	
**Listening to the radio**							
Notat all	253,236(68.6)	68.1	<0.001	22.8	0.067	2983.8(2980.4, 2987.2)	<0.001^***^
< once a week	50,288(13.6)	71.6		19.8		3083.5(3076.3, 3090.8)	
At least once a week	65,511(17.8)	73.0		17.8		3191.0(3185.2, 3198.6)	
**Reading newspaper**							
Notat all	278,345(75.4)	67.6	<0.001	18.59	<0.001	3039(3035.7, 3042.2)	<0.001^***^
< once a week	54,458(14.8)	73.8		25.3		3004.6(2997.6, 3011.5)	
At least once a week	36,232(9.8)	77.3		30.3		3043.4(3034.3, 3052.5)	
**Toilet facility**							
Improved	252,686(68.5)	72.3	<0.001	24.0	<0.001	3032.4(3028.6, 3035.5)	<0.001^***^
Unimproved	25,444(6.9)	71.1		22.8		3021.9(3011.5, 3032.4)	
Open defecation	90,904(24.6)	60.9		11.0		3044.0(3038.6, 3049.4)	

p-value* for chi-square test.

P-value** for an independent t-test.

p-value *** for one-way ANOVA.

### The magnitude of low birth weight

#### Weighted prevalence of low birth weight.

**Fig 4:**
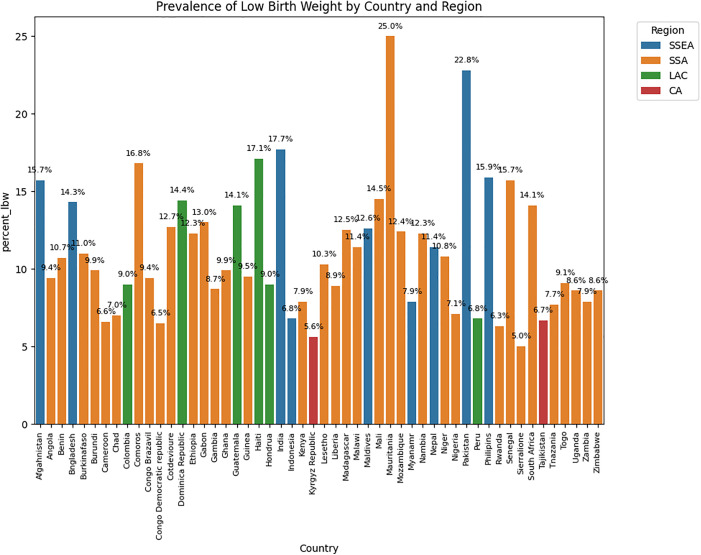
Distribution of low birth weight across Low- and middle-income countries, 2025.

The weighted prevalence of LBW was 12.42% (95% CI: 12.31, 12.52), with a mean birth weight of 3034.31 (±651.65) grams. The highest prevalence was recorded in Mauritania (25.0%), Pakistan(22.8%), India (17.7%), and Haiti (17.1%). On the other hand, the lowest prevalence was reported in Sierra Leone (5.0%). Regional disparities were clear (p < 0.005), with the highest prevalence seen in South and Southeast Asia and Central Asia regions at 15.7% and 5.4%, respectively (Fig 4).

### Estimation of propensity scores of treatments

To estimate the propensity scores for receiving antenatal care treatments—defined as ≥4 ANC visits (Treatment 1) and ≥8 ANC visits (Treatment 2), a logit regression model was employed using a range of maternal, socioeconomic, and contextual variables. The variables that have a significant association with both treatments (ANC8+ and ANC4+) and outcome (birthweight) were considered for matching using the logit model. These coefficients were used to compute individual-level propensity scores for each treatment group, representing the predicted probability of receiving ANC4+ or ANC8+ based on covariate profiles (Table 4).

**Table 4:** Results of logit regression analysis of factors associated with treatments (ANC4+ and ANC8+) in LMICs, 2025.

Variables	Treatment 1 (ANC4+)		Treatment 2 (ANC8+)	
	Coefficient	p-value	Coefficient	p-value
**Region**	0.276889	<0.001	-0.23735	<0.001
Maternal age	0.173394	<0.001	0.327133	<0.001
Level of education	0.245885	<0.001	0.332398	<0.001
Residence	-0.38556	<0.001	-0.52064	<0.001
Wealth index	-0.02063	<0.001	0.00225	0.597
Parity	-0.11265	<0.001	-0.13662	<0.001
ANC timing	-1.44025	<0.001	-1.35169	<0.001
Planning status of pregnancy	0.014268	0.039	0.130962	<0.001
Contraceptive uptake	0.177422	<0.001	0.116468	<0.001
Ease of getting money for health care	-0.15177	<0.001	-0.39858	<0.001
Permission to go to the health facility	-0.26481	<0.001	-0.33723	<0.001
Listening to the radio	0.024584	<0.001	0.120732	<0.001
Watching TV	0.042728	<0.001	-0.03532	<0.001
Reading newspaper	-0.0139	0.044	-0.0021	0.773
Enrolled in Health insurance	-.0194614	0.045	-0.01434	0.191
Autonomy in decision making			-0.14163	<0.001
_cons	2.473539	_cons	1.573043	<0.001

### Distribution of propensity scores for treatments

**Fig 5:**
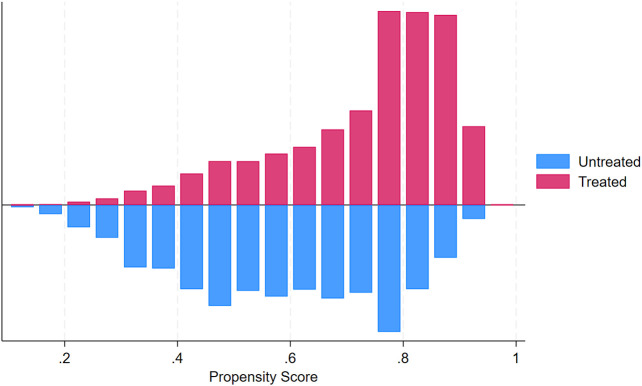
Histogram showing the distribution of estimated propensity scores by treatment status (ANC4+ vs. fewer visits).

**Fig 6:**
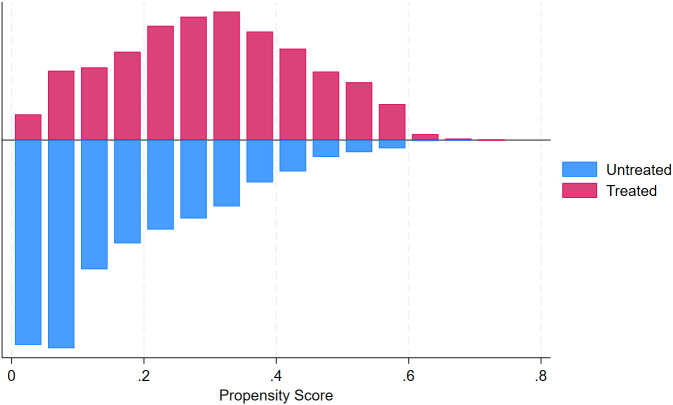
Histogram showing the distribution of estimated propensity scores by treatment status (ANC8+ vs. fewer visits).

The distribution of propensity scores for receiving treatments (ANC4+ and ANC8+) shows a distinct separation between women who received the treatment and those who did not. This indicates that the logistic regression model used to estimate treatment likelihood performed well in distinguishing between the two groups. For ANC4+ (Treatment 1), the average propensity score was 0.689, meaning that, based on their characteristics, women in the sample had a 68.9% predicted probability of receiving this level of care. This is visually supported by the histogram, which shows a higher concentration of treated individuals at the upper end of the score range in (Fig 5). The average propensity score for receiving ≥8 ANC visits (ANC8+, Treatment 2) was much lower at 0.19, suggesting that women in the study population had only a 19% predicted probability of receiving this more intensive care. This lower score aligns with the actual treatment rate, as only 20.73% of women received ANC8+ (Fig 6). Overall, both histograms show overlap between the two groups in the middle range of scores, which ensures that treated and untreated individuals share similar characteristics, allowing for fair comparisons using propensity score matching.

### The causal effect of adequate ANC (ANC4+) on birth weight

The matched estimates provide robust evidence that both ANC4+ and ANC8+ are associated with significant gains in birth weight, with ANC8+ showing a stronger effect among those who received the treatment. Table 5 presents the results of a propensity score-matched analysis assessing the effect of two ANC models, ANC4+ and ANC8+, on birth weight across LMICs. The analysis includes estimates for unmatched samples, as well as matched estimates for the Average Treatment Effect on the Treated (ATT), the Untreated (ATU), and the overall population (ATE). For ANC4+, the unmatched analysis shows a substantial mean birth weight difference of 90.38 grams between treated and untreated. After matching, the ATT reveals that women who received ANC4+ had babies with a mean birth weight 76.414 grams higher than their matched counterparts who did not receive the treatment. The overall treatment effect across the population (ATE) is 70.94 grams. For ANC8+, the unmatched difference in birth weight is 122.411 grams, indicating a statistically significant effect on birth weight compared to ANC4+. The matched ATT shows a more pronounced impact; treated women had babies weighing 104.157 grams more than matched controls (untreated). The ATE for the entire population is 84.60 grams (Table 5).

**Table 5:** A propensity score-matched analysis of the impact of Treatments (ANC4+ and ANC8+) on birth weight in LMIC countries, 2025.

Treatments	Sample	Treated	Controls	Difference (mean)	S.E.	T-stat
ANC4+	Unmatched	3069.840	2979.457	90.383	2.296135	39.36
	ATT	3069.840	2993.426	76.414	14.0976199	5.42
	ATU	3039.640	2979.456	60.184	.	.
	ATE			70.9405		
ANC8+	Unmatched	3067.840	2945.429	122.411	3.687626	42.06
	ATT	3067.840	2963.683	104.157	23.24673	4.85
	ATU	3035.429	2962.875	72.554	.	.
	ATE			84.605	.	.

Key: ATT: Average Treatment Effect among Treated, ATU: Average Treatment Effect on Untreated, ATE: Average Treatment Effect on the whole population.

## Quality of matching

### Standard bias and model significance

The ability of the matching procedure to balance the distribution of relevant covariates between the treatment and control groups was evaluated. The pseudo-R^2^ statistic was used to assess how well the covariates explained the likelihood of treatment assignment. For the first treatment (ANC8+), the pseudo-R^2^ declined markedly from 0.134 in the unmatched sample to 0.001 post-matching, indicating a significant reduction in covariate imbalance between treatment and control groups. Additionally, the mean standardized bias decreased from 25.6% to 1.7%, while the median bias reduced from 19.1% to 01.4%, collectively demonstrating substantial improvement in covariate balance and suggesting that the matching procedure was successful. For the other treatment (ANC4+), the pseudo-R^2^ value decreased markedly from 0.11 before matching to 0.001 post-matching, while the maximum absolute standardized bias (B) dropped from 84.8% to 7.3%, indicating a significant improvement in covariate balance (Table 6).

**Table 6:** Performance of the propensity score matching for the effect of ANC8+ and ANC4+ on birthweight in LMICs, 2025.

Intervention	Sample	Ps R2	LR chi2	p>chi2	MeanBias	MedBias	B	R*	%Var
ANC4+	Unmatched	0.119	54559.25	0.000	23.4	18.4	87.0*	0.89	100
	Matched	0.00	233.12	0.00	0.8	0.6	4.3	1.07	36
ANC8+	Unmatched	0.139	50818.63	0.000	29.4	23.9	100.6*	0.61	100
	Matched	0.000	36.93	0.001	0.8	0.6	3.2	1.07	31

* Ratio of Treated vs Untreated group.

### Balancing test

The pstest command in Stata was employed to assess covariate balance between treated and control groups before and after matching. Before matching, most covariates exhibited substantial bias. Differences between unmatched and matched samples were assessed using t-tests. The t-tests indicated there was a significant mean difference for each covariate before matching (p < 0.05), but after matching, the percentage bias for all covariates was reduced, and the results indicated no significant mean differences across nearly all factors (p > 0.05). In both cases, the matching process decreased the bias by more than 95% for each covariate. This demonstrates that the variables were adequately balanced (Tables 7 and 8).

**Table 7:** Quality of matching for the effect of ANC4+ on birthweight in LMCs, 2025.

Variable		Mean		%bias	Bias reduction	T-test	
		Treated	Control			T_statstics	p-value
Region	Unmatched	2.2963	2.2018	6.7		18.99	<0.001
	Matched	2.2963	2.2949	0.1	98.5	0.37	0.714
Level of education	Unmatched	1.6225	1.3122	33.4		94.84	<0.001
	Matched	1.6225	1.6107	1.3	96.2	4.60	0.234
Residence	Unmatched	1.6122	1.7487	-29.6		-81.62	<0.001
	Matched	1.6122	1.6102	0.4	98.5	1.51	0.330
Wealth index	Unmatched	3.0353	2.6577	27.2		76.39	<0.001
	Matched	3.0353	3.0479	-0.9	96.7	-3.20	0.431
Parity	Unmatched	2.4207	2.5646	-13.6		-38.25	<0.001
	Matched	2.4207	2.4155	0.5	96.4	1.74	0.682
Contraceptive uptake	Unmatched	.54743	0.48254	13.0		36.69	<0.001
	Matched	.54743	0.54493	0.5	96.1	1.79	0.573
ANC timing	Unmatched	1.2843	1.6016	-67.4		-192.91	<0.001
	Matched	1.2843	1.2873	-0.6	99.1	-2.33	0.328
Accessing money for health care	Unmatched	1.2879	1.3743	-18.4		-52.64	<0.001
	Matched	1.2879	1.2849	0.6	96.6	2.35	0.419
Permission to go to the health facility	Unmatched	1.1215	1.1864	-18.0		-52.62	<0.001
	Matched	1.1215	1.117	1.2	93.1	4.94	0.368
Watching TV	Unmatched	1.0276	0.85223	19.3		53.97	<0.001
	Matched	1.0276	1.0265	0.1	99.3	0.44	0.657
Reading newspaper	Unmatched	.35502	0.25941	15.6		42.74	<0.001
	Matched	0.35502	0.3495	0.9	94.2	3.02	0.633

**Table 8:** Quality of matching for the effect of ANC8+ on birthweight in the LMICs, 2025.

Variable		Mean		%bias	Bias reduction	T-test	
		Treated	Control			T-statstics	p-value
Region	Unmatched	1.7518	2.3918	-50.0		-111.87	0.000
	Matched	1.7518	1.7489	0.2	99.6	0.50	0.618
Maternal age	Unmatched	1.924	1.8455	11.7		27.87	0.000
	Matched	1.924	1.9208	0.5	95.9	0.92	0.358
Level of education	Unmatched	1.9131	1.4322	54.2		127.01	0.000
	Matched	1.9131	1.9099	0.4	99.3	0.74	0.460
Residence	Unmatched	1.5311	1.6846	-31.8		-78.51	0.000
	Matched	1.5311	1.5319	-0.2	99.5	-0.31	0.760
Wealth index	Unmatched	3.2504	2.8373	29.8		71.46	0.000
	Matched	3.2504	3.2485	0.1	99.5	0.26	0.795
Parity	Unmatched	2.2834	2.5096	-21.5		-51.53	0.000
	Matched	2.2834	2.2846	-0.1	99.5	-0.21	0.830
ANC timing	Unmatched	1.1326	1.4436	-73.1		-159.56	0.000
	Matched	1.1326	1.1327	-0.0	100.0	-0.09	0.932
Contraceptive uptake	Unmatched	.60939	.50734	20.7		49.46	0.000
	Matched	.60939	.61125	-0.4	98.2	-0.72	0.470
Perceived distance to health facility	Unmatched	1.1539	1.2921	-33.7		-76.08	0.000
	Matched	1.1539	1.1465	1.8	94.6	3.94	0.328
Accessing money for health care	Unmatched	1.1657	1.3509	-43.3		-97.41	0.000
	Matched	1.1657	1.1564	2.2	95.0	4.82	0.632
Permission to go to the health facility	Unmatched	1.0785	1.157	-24.6		-54.55	0.000
	Matched	1.0785	1.0738	1.5	94.0	3.37	0.537
Watching TV	Unmatched	1.1498	.93029	23.9		57.85	0.000
	Matched	1.1498	1.137	1.4	96.1	2.65	0.338
Autonomy in decision making	Unmatched	1.7982	1.7292	16.3		38.10	0.000
	Matched	1.7982	1.8019	-0.9	94.6	-1.76	0.078
Enrolment in health insurance	Unmatched	.23416	.18892	11.1		27.42	0.000
	Matched	.23416	.22487	2.3	96.5	4.20	0.000

### Common support assumption

The common support assumption was assessed graphically and which revealed substantial overlap in the distribution of propensity scores between treated and control groups, substantiating the validity of the common support assumption. Visual inspection of the graph for ANC4+ revealed the propensity score of both treated and control groups concentrated between 0.4 and 0.8. This pattern suggests that a majority of individuals had a good predicted probability of receiving ANC4+, and substantial overlap was evident, supporting the presence of common support within the matched sample (Fig 5). Visual inspection of the graph for ANC8+ revealed a markedly skewed distribution of estimated propensity scores, with both treated and control groups concentrated at the lower end of the scale (0–0.3) and tapering off toward higher values. This pattern suggests that a majority of individuals had a low predicted probability of receiving ANC8+, yet substantial overlap was still evident, supporting the presence of common support within the matched sample (Fig 6).

### Post-matching covariate balance plot

**Fig 7:**
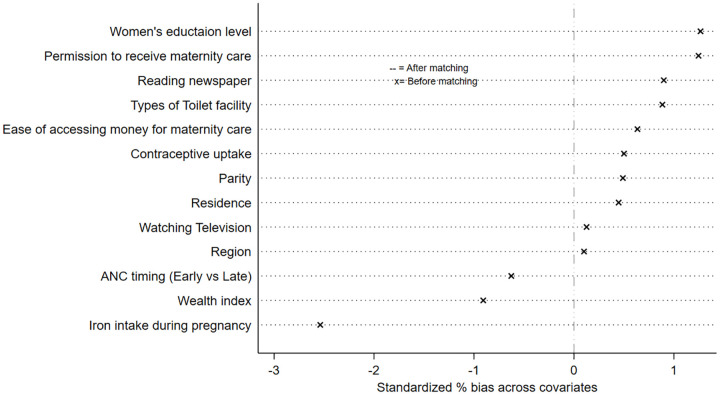
Standardized percentage bias in the distribution of confounders before and after matching for ANC4+.

**Fig 8:**
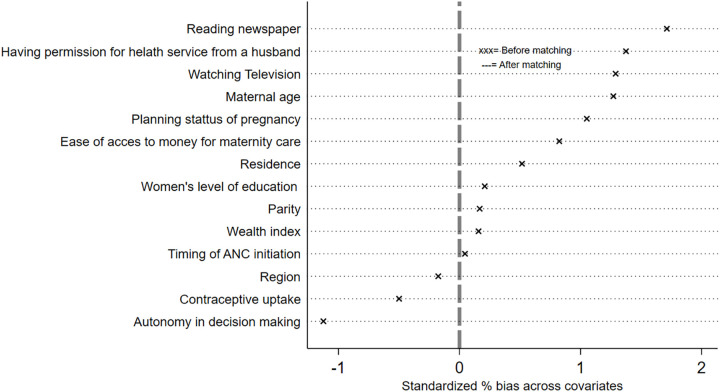
Standardized percentage bias in the distribution of confounders before and after matching for ANC8+.

To evaluate the effectiveness of the PSM procedure for women who received eight or more antenatal care (ANC8+) visits, covariate balance was assessed using standardized percentage bias plots. This visual diagnostic compared pre- and post-matching standardized biases across key covariates. The resulting graph demonstrated a marked reduction in bias, with all post-matching values tightly clustered around zero and falling well within the conventional ±10% threshold. These findings confirm that the matching algorithm successfully eliminated systematic differences between treatment and control groups across observed covariates, thereby enhancing the validity of subsequent treatment effect estimates for ANC8+ on birthweight outcomes (Fig 7). Similarly, a covariate balance was assessed for ANC4+, and the resulting balance plot demonstrated a marked reduction in standardized mean differences for all included covariates, with post-matching values falling well within the ±10% threshold(−6 to 2). This indicates that the matching procedure successfully mitigated baseline differences, supporting the validity of subsequent treatment effect estimates on birthweight (Fig 8).

### Sensitivity analysis

In the presence of unobserved variables exerting simultaneous influence on both the assignment into exposure and the outcome variable, the emergence of hidden bias becomes a concern. To handle this, the Rosenbaum bounding method was employed (as the outcome is a continuous variable) to ascertain the extent to which unmeasured variables, or hidden bias, impact the selection process and, consequently, the implications of the matching analysis. Strong evidence that ANC8+ and ANC4+ increase neonatal birthweight would be found in all of the analyses, in a study free of bias, that is, where Ґ = 1. The upper bound on the significance level for Ґ = 1.05, 1.1, 1.15……2. The final output demonstrated consistent robustness of the estimated treatment effects across both treatment groups (ANC8+ and ANC4+). For each specification, the upper and lower bounds remained statistically significant across a range of Γ values, suggesting that the observed associations are unlikely to be explained by unmeasured confounding, which enhances the credibility of the findings (Tables 9 and 10).

**Table 10:** Sensitivity analysis using Rosenbaum bounds for the effect of ANC8+ on birthweight.

Gamma	sig+	sig-	t-hat+	t-hat-	CI+	CI-
1	0	0	3049	3049	3024	3049
1.1	0	0	2999	3049	2999	3054
1.2	0	0	2999	3099	2999	3099
1.3	0	0	2999	3099	2999	3099
1.4	0	0	2949	3132	2949	3149
1.5	0	0	2949	3149	2949	3149
1.6	0	0	2929	3149	2899	3174
1.7	0	0	2899	3199	2899	3199
1.8	0	0	2899	3199	2899	3199
1.9	0	0	2899	3214	2874	3236.5
2	0	0	2849	3249	2849	3249

gamma - log odds of differential assignment due to unobserved factors.

sig+ - upper bound significance level.

sig- - lower bound significance level.

t-hat+ - upper bound Hodges-Lehmann point estimate.

t-hat- - lower bound Hodges-Lehmann point estimate.

CI+ - upper bound confidence interval (a=.95).

CI- - lower bound confidence interval (a=.95).

**Table 9:** Sensitivity analysis using Rosenbaum bounds for birthweight for ANC8+ in LMICs, 2025.

Gamma	sig+	sig-	t-hat+	t-hat-	CI+	CI-
1	0	0	3000	3000	3000	3000
1.1	0	0	3000	3050	3000	3050
1.2	0	0	3000	3050	3000	3060
1.3	0	0	2975	3100	2966	3100
1.4	0	0	2950	3100	2950	3100
1.5	0	0	2929	3125	2925	3130
1.6	0	0	2900	3150	2900	3150
1.7	0	0	2900	3150	2900	3150
1.8	0	0	2875	3200	2875	3200
1.9	0	0	2850	3200	2850	3200
2	0	0	2850	3201.5	2850	3213

gamma - log odds of differential assignment due to unobserved factors.

sig+ - upper bound significance level.

sig- - lower bound significance level.

t-hat+ - upper bound Hodges-Lehmann point estimate.

t-hat- - lower bound Hodges-Lehmann point estimate.

CI+ - upper bound confidence interval (a=.95).

CI- - lower bound confidence interval (a=.95).

## Discussion

Our findings underscore the critical role of ANC intensity in improving birth outcomes across LMICs. The weighted prevalence of LBW was 12.42% (95% CI: 12.31, 12.52), indicating that LBW remains a substantial public health challenge in these settings despite ongoing maternal and child health interventions. Furthermore, only 69.45% (95% CI: 69.29, 69.59) and 20.73% (95% CI: 20.59, 20.89) of women achieved the WHO-recommended thresholds of at least four (the former) and eight (the current) ANC visits, respectively, highlighting persistent gaps in the utilization of maternal health services. Using PSM to minimize selection bias, we found that both ANC4+ and ANC8 + models were significantly associated with increased birthweight, with ANC8+ demonstrating a larger effect size.

The weighted prevalence of low birthweight (LBW) in this study was 12.42%, which is lower than recent global estimates reported by UNICEF (14.7%) [5], Hannah Blencowe et al. (13.7%) [65], and Okwaraji et al. (14.7%) [4], Asian countries (16.64%) [66], ten developing countries (15.9%) [40]. However, it exceeds estimates reported from Sub-Saharan Africa, including (10.44%) [67] and 9.76% [25], and a recent estimate from 14 West African countries (10.42%) [68]. These variations may reflect differences in the geographical composition of study populations, socioeconomic and healthcare contexts, maternal nutritional status, and access to maternal health services. Furthermore, disparities in study periods, sample sizes, and methodological approaches, including the source of birthweight data (health card records versus maternal recall), may contribute to the observed differences. The relatively lower prevalence observed in our study may also reflect gradual improvements in maternal and child health interventions across many LMICs, including expanded access to ANC, nutritional supplementation, and strengthened health systems [69,70]. Nevertheless, the persistence of LBW in more than one in ten births underscores the continuing challenge of achieving optimal fetal growth and highlights the need for sustained investment in maternal healthcare services, particularly in resource-constrained settings.

Women who received ANC4+ had newborns weighing 76.41 grams more than matched controls, while ANC8+ was associated with a greater increase of 104.16 grams. These findings are consistent with previous studies reporting positive effects of antenatal care utilization on birthweight [33,68,71,72]. For instance, a population-based study conducted in The Gambia demonstrated that each additional ANC visit was associated with a 22-gram increase in birthweight [71]. Likewise, substantial evidence indicates that adherence to recommended ANC schedules reduces the risk of LBW and other adverse neonatal outcomes, highlighting the critical role of sustained maternal healthcare throughout pregnancy [34,37,73–75]. The observed associations may reflect the cumulative benefits of ANC in addressing major maternal risk factors for LBW, including anaemia, infections, hypertensive disorders, and nutritional deficiencies, through repeated opportunities for risk assessment, health education, nutritional counselling and supplementation, preventive interventions, and timely referral for specialized care [76–79]. Importantly, the larger effect observed among women receiving ANC8+ compared with ANC4+ suggests a dose-response relationship between ANC utilization and fetal growth, whereby increasing contact with skilled healthcare providers confers additional benefits for maternal and neonatal health. This finding lends empirical support to the WHO recommendation of a minimum of eight ANC contacts [52] and indicates that the benefits of ANC extend beyond service utilization alone to encompass continuity and quality of care. Although the observed increases in birthweight may appear modest at the individual level, even small upward shifts in the population distribution of birthweight can substantially reduce the prevalence of low birthweight and its associated risks of neonatal morbidity, mortality, impaired growth, and long-term developmental consequences. Nevertheless, the suboptimal uptake of ANC4+ (69.45%) and particularly ANC8+ (20.73%) identified in this study highlights a considerable gap in achieving recommended ANC coverage across LMICs. Given the significant positive effect of ANC on birthweight, strengthening access to, utilization of, and continuity of high-quality ANC services should remain a priority public health strategy for improving neonatal outcomes and accelerating progress toward global maternal and child health targets [80,81].

### Policy and practice implications

The findings of this study carry important policy and practice implications for improving maternal and neonatal health outcomes in LMICs. The significant positive association between ANC utilization and birthweight, coupled with the stronger effect observed among women receiving ANC8+, highlights the importance of ensuring not only access to ANC services but also continuity of care throughout pregnancy. Given that only 69.45% and 20.73% of women achieved the recommended ANC4+ and ANC8+ contacts, respectively, substantial opportunities remain to improve maternal healthcare utilization. From a practice perspective, healthcare providers should prioritize early ANC initiation, promote adherence to recommended ANC schedules, and ensure the delivery of essential evidence-based interventions during each contact, including nutritional counselling and supplementation, screening and management of maternal infections, and health education. Strengthening the quality and content of ANC services may maximize the benefits of increased contact frequency and further improve fetal growth outcomes.

From a policy perspective, the findings support accelerated implementation of the newly WHO-recommended ANC8+ model across LMICs [36]. Policymakers should invest in strengthening health system infrastructure, expanding the maternal health workforce, reducing financial and geographical barriers to care, and implementing community-based strategies that encourage ANC attendance. Particular attention should be given to Sub-Saharan Africa, where ANC8+ coverage remains critically low. Expanding access to comprehensive ANC, especially in line with the WHO-recommended ANC8+ contact model, presents a high-impact opportunity to further reduce LBW and ultimately advance maternal and neonatal health equity.

### Strengths and limitations

This study demonstrates several methodological and contextual strengths. The use of PSM effectively reduced selection bias commonly associated with observational data, thereby strengthening the validity of causal inferences regarding the effect of adequate ANC on birthweight. By estimating both the average treatment effect on the treated (ATT) and the average treatment effect (ATE), the study provides a comprehensive assessment of the impact of ANC utilization on birthweight among women receiving the interventions and across the broader population. Furthermore, the large, nationally representative sample drawn from 52 LMICs enhances the generalizability of the findings. The robustness of the analysis was further supported by extensive balance diagnostics, which confirmed the adequacy of the matching procedure and the comparability of treatment groups.

However, some limitations should be considered when interpreting the findings. First, although DHS surveys employ standardized questionnaires, sampling procedures, and data collection protocols, differences in healthcare systems, sociocultural contexts, and survey implementation across the 52 countries may affect the comparability of data and limit cross-country uniformity. Second, residual confounding from unmeasured factors, such as maternal nutritional status, environmental exposures, genetic influences, and healthcare quality, cannot be entirely ruled out because PSM accounts only for observed covariates. Third, birthweight data were obtained from multiple approaches (i.e., health cards/medical records or maternal recall, depending on data availability within each survey), which may have introduced measurement error. Finally, the cross-sectional nature of DHS data and reliance on maternally reported information may be subject to recall bias and social desirability bias, particularly for birthweight records obtained from maternal recall rather than documented health records. These limitations should be considered when interpreting the magnitude of the estimated treatment effects.

## Conclusion

The prevalence of LBW (12.42%) observed in this study highlights the persistent burden of suboptimal fetal growth across LMICs. Using propensity score matching, we found that both ANC4+ and ANC8+ were associated with significant improvements in birthweight, with the larger effect observed among women receiving ANC8+, suggesting a dose-dependent relationship between ANC utilization and fetal growth. These findings provide empirical support for the WHO recommendation of a minimum of eight ANC contacts during pregnancy.

Given the substantial benefits observed and the low coverage of ANC8+ (20.73%), policymakers and healthcare systems should prioritize strategies that improve both access to and continuity of high-quality ANC services by promoting early ANC initiation.

## Abbreviation:

ANC: antenatal care
ATT: average treatment effect on the treated
ATE: average treatment effect
DHS: demographic and health survey
LBW: low birthweight
LMICs: low- and middle-income countries
PSM: propensity score matching.
