# Supplementary material for: Estimating the effect of antenatal care models on birthweight in low- and middle-income countries: Evidence from a DHS-based propensity score matching analysis
Source: PLoS One. 2026 Aug 14;21(8):e0355685. doi: 10.1371/journal.pone.0355685 (PMC13475944; doi:10.1371/journal.pone.0355685)
Supplement: Supplementary file 1 [file pone.0355685.s001.docx]

| Title and abstract |  |
| --- | --- |
| Introduction | Major comment: Please elaborate about the burden of LBW in LMICs  Minor comment: Since COVID-19 is no more a public health concern, it might not be implacable to be included in the study. |
| Methods | Preparing a flow diagram to explain sampling would be deme  How were the missing data for other than birthweight, ANC  records, and multiple births handled? |
| Other Information | Please mention line number, which will make further review easier |
