# Supplementary material for: Estimating the effect of antenatal care models on birthweight in low- and middle-income countries: Evidence from a DHS-based propensity score matching analysis
Source: PLoS One. 2026 Aug 14;21(8):e0355685. doi: 10.1371/journal.pone.0355685 (PMC13475944; doi:10.1371/journal.pone.0355685)
Supplement: Supplementary file 2 [file pone.0355685.s002.docx]

**A point-by-point response to the editor and reviewer**

**Dear Dr Sabita Tuladhar, PhD, MHealSc, MA, Academic Editor**

Thank you for your careful evaluation of our manuscript and for providing us with the opportunity to revise and resubmit our work. We are pleased to submit the revised version of our manuscript, entitled **“Estimating the Effect of Antenatal Care Models on Birthweight in Low- and Middle-Income Countries: Evidence from a DHS-Based Propensity Score Matching analysis” (Manuscript ID: PONE-D-26-04747)**.

We sincerely appreciate the insightful and constructive comments provided by you and the reviewers. The feedback has been invaluable in strengthening the scientific rigor, methodological robustness, and overall presentation of the manuscript. In response, we have undertaken some revisions throughout the manuscript.

All comments have been addressed in a detailed point-by-point response document. For ease of evaluation, they have been highlighted throughout the revised manuscript using track changes, and the corresponding page and line numbers are provided in our responses to the reviewer’s comments.

We are grateful for the time and effort invested by the editor and a reviewer in improving our manuscript. We hope that the revisions adequately address all concerns and that the manuscript is now suitable for publication in PLOS ONE. We remain happy to consider any additional suggestions that may further strengthen the manuscript.

On the following pages, we provide our detailed responses to the reviewer’s comments.

With best regards,

Aklilu Habte Hailegebireal, MPH

Corresponding Author

[akliluhabte57@gmail.com](mailto:akliluhabte57@gmail.com)

**Response to the additional editor’s comment**

**Comment:** The study included women aged 15-49 years who had a live birth within five years preceding the survey. Pls confirm if the reference period was five, three or two years. It should be clarified whether all DHS surveys consistently measured the variables of interest across the 52 countries.

**Response:** Thank you for this important comment. We have clarified the reference period and the availability of study variables across the included DHS surveys. The analysis was restricted to women aged 15-49 years who had a live birth within the five years preceding each survey, consistent with the standard DHS birth recode dataset. We further verified that the key variables required for this study, including antenatal care utilization, birthweight, maternal sociodemographic characteristics, and other covariates, were available and measured using standardized DHS questionnaires across all 52 included countries. To ensure comparability, only surveys containing the variables necessary for defining antenatal care models, birthweight outcomes, and matching covariates were included in the analysis. We tried to make it clearer and highlighted in the method section of the ‘Revised manuscript with track changes’, Page 4, Line 122-128.

Comment 2: Clarify how birthweights were obtained across DHS surveys: Were they measured using standardized weighing scales? Or based on maternal report (recall or health card)? Differences in measurement methods should be explicitly stated.

**Response:** Thank you for this important observation. We have clarified the source and measurement of birthweight in the Methods section. In DHS surveys, birthweight is not measured directly by DHS interviewers. Instead, birthweight information is obtained either from the child’s health card or medical record when available, or from maternal recall when documentary records are unavailable. As measurement methods may vary across settings, we have acknowledged this as a potential source of measurement error and discussed it as a limitation of the study. Please make a look over the method section of the ‘Revised manuscript with track changes’, Page 7, Line 158-162.

Comment 3: Additional limitations should be acknowledged as applicable, including:

- Comparability of data across the 52 countries
- Variations in birthweight measurement approaches
- Potential recall bias in maternally reported birthweights

**Response:** Thank you for this valuable comment. We agree that these are important limitations that should be acknowledged. Accordingly, we have expanded the limitations section to discuss potential issues related to data comparability across the 52 included countries, variations in birthweight measurement approaches (health card/medical record versus maternal recall), and the possibility of recall bias in maternally reported birthweights. These additions have been incorporated and highlighted in the Strengths and limitations section of the ‘Revised manuscript with track changes’, Page 24, Lines 604-616.

**Authors’ Response to Reviewer**#**1**

**General comment:** Thank you for your nice work. I really enjoyed. The manuscript is clearly written. However, the authors need to address the following points:

**Response:** We sincerely appreciate your positive evaluation of our manuscript and your encouraging comments regarding its clarity and overall quality. We appreciate the time and effort invested in reviewing our work. We have carefully considered all comments and suggestions and have revised the manuscript accordingly. Detailed responses to each point are provided below, and all corresponding changes have been highlighted in the revised manuscript with track changes.

**Comment 1:** The discussion is superficial. The authors need a high discussion.

**Response:** Thank you for this constructive comment. Accordingly, we thoroughly revised and expanded the Discussion to provide a more comprehensive interpretation of the findings, compare our results with existing evidence from low- and middle-income countries and global literature, explore potential mechanisms underlying the observed associations, and discuss the public health and policy implications of adequate antenatal care utilization. We have also strengthened the discussion of the study's strengths and limitations. These revisions have substantially enhanced the depth and scholarly contribution of the Discussion section and are highlighted in the revised manuscript with track changes.

**Comment 2:** Implication for practice is missing.

**Response:** Thank you for this valuable observation. We agree that the implications for practice were not sufficiently explicit in the previous version of the manuscript. Accordingly, we have revised and expanded this section to clear out the practical and policy implications of our findings. Specifically, we highlighted the importance of strengthening access to and continuity of antenatal care services, promoting adherence to the WHO-recommended ANC8+ model, and prioritizing interventions that improve ANC utilization and quality of care in low- and middle-income countries. These revisions have been incorporated and highlighted in the Discussion section of the ‘revised manuscript with track changes’, Page 23, Line 577-591.

**Comment 3:** Recommendation part needs retuning

**Response:** Thank you for this constructive comment. We agree that the recommendation section requires further refinement to better reflect the study findings and their implications for policy and practice. Accordingly, we revised the recommendations to provide more specific, actionable, and evidence-informed guidance for policymakers, healthcare providers, and maternal health programs. The revised version is highlighted in the ‘conclusion’ section of the ‘revised manuscript with track changes’, Page 24, Line 618-626.

Thank you once again for your valuable time, thoughtful review, and important contributions to improving our manuscript.

**_________________________________________________________________________________________________________END_______________________________________**

**THANK YOU!!!**

**Response to Reviewer #2 (Shristi Rijal) comment**

Dear Shristi Rijal, we sincerely appreciate your positive evaluation of our manuscript and your encouraging comments regarding its clarity and overall quality. We appreciate the time and effort invested in reviewing our work.

**Comment 1:** Introduction Major comment: Please elaborate about the burden of LBW in LMICs

**Response:** Thank you for this valuable comment. We agree that the burden of low birthweight (LBW) in LMICs requires further elaboration. Accordingly, we expanded the Introduction to provide additional context on the magnitude of LBW in LMICs, its contribution to neonatal morbidity and mortality, adverse childhood growth and developmental outcomes, and its long-term health and socioeconomic consequences. These were highlighted in the ‘background’ section of the ‘revised manuscript with track changes’, Page 3, Line 68-78.

**Comment 2:** Preparing a flow diagram to explain sampling would be deme

**Response:** Thank you for this valuable suggestion. We agree that a flow diagram would improve the transparency of the sampling process and participant selection. Accordingly, we have developed and incorporated a detailed flow diagram **(Figure 1)** illustrating the sampling procedure, including the initial pooled DHS sample, application of eligibility criteria, exclusions, derivation of the final analytical sample, and classification of participants into the ANC4+ and ANC8+ exposure groups for propensity score matching analyses. The sampling procedure has been further elaborated in the “Sampling Techniques and Procedures” section of the ‘revised manuscript with track changes’ (Page 6, Lines 138-151).

**Comment 3:** How were the missing data for other than birthweight, ANC

records, and multiple births handled?

**Response:** Missing data were assessed for all study variables prior to analysis. Records with missing information on key variables, including birth weight, antenatal care (ANC) attendance, and plurality, were excluded because these variables were essential for defining the study population and conducting the planned analyses. For all other variables, the proportion of missing data was low (<2% in this scenario) and was therefore retained as missing. Analyses were conducted using complete-case observations for the variables included in each statistical model. No imputation procedures were performed, as the level of missingness was considered unlikely to affect the study findings.

We sincerely thank you for your thoughtful, constructive, and insightful comments provided on our manuscript. We greatly appreciate the time and effort invested in reviewing our work. All corresponding revisions have been incorporated and highlighted in the revised manuscript with track changes, with the relevant page and line numbers indicated for ease of reference.

**_________________________________________________________________________________________________________END_______________________________________**

**THANK YOU!!!**
