# Supplementary material for: Estimating the effect of antenatal care models on birthweight in low- and middle-income countries: Evidence from a DHS-based propensity score matching analysis
Source: PLoS One. 2026 Aug 14;21(8):e0355685. doi: 10.1371/journal.pone.0355685 (PMC13475944; doi:10.1371/journal.pone.0355685)
Supplement: Supplementary file 3 [file pone.0355685.s003.docx]

**Reviewer Feedback**

**1. Introduction section**

Comment: Statement on line 77 ("20 times higher mortality risk") is stark—ensure this is directly cited. Reference [2, 3] provided; confirm source is clear.

**Gap noted:** Lines 98–101 repeat the evidence gap statement. Lines 95–101 are somewhat redundant. Consider consolidating to: "Although LBW burden in LMICs is well-studied, limited evidence exists comparing effectiveness of ANC4+ versus WHO-recommended ANC8+ models using rigorous causal methods.”

**Suggestion:** Line 117–118 mentions 30% reduction target "extended to 2030"—confirm this is accurate and cite appropriately.

**2. Methods**

**Line 129:** Final sample "369,034 women from 52 LMICs (Table 1)": Sample size is substantial

**Data issue (minor):** Table 1 footer shows total as "370,579" but text says "370,576" (line 142). Confirm which correct unweighted N. is this discrepancy should be resolved for publication.

**Lines 173–178:** Clear definition of ANC4+ and ANC8+ with reference to WHO recommendations [36, 50–52]. Good.

**Question:** Were women with 0 ANC visits included in analysis? This is clinically important but not explicitly stated. Clarify whether control group includes women with any ANC or those with none.

**Parity definition:** Line 206–207 defines parity as "number of living children." Is this the same as DHS definition? Should confirm for clarity.

**Lines 228–232:** Bivariate analysis approach for confounder identification is appropriate. Chi-square and t-tests as described in Statistics section (line 245–250)

**Lines 330–337:** Key findings presented: Was analysis conducted stratified by region? Sub-Saharan Africa has critically low ANC8+ coverage (8.9%). Separate models for high vs. low ANC8+ uptake regions could strengthen regional applicability.

**Line 244–245:** Use of DHS sampling weights to ensure nationally representative estimates—excellent. This is crucial for generalizability.

**Table 3 comment:** Line 364 notes parity differences significant (p<0.001). The pattern of birthweight by parity is expected (grand multiparity associated with increased parity-related risks in some contexts but higher birthweight in others). Discussion should address this.

**3. Discussion Section**

**Lines 526–541 (LBW prevalence context):** Compares study prevalence (12.42%) with global estimates (UNICEF 14.7%, others 13.7%–16.64%). Notes variations likely due to geographic, socioeconomic, methodological differences

**Comment:** Authors mention "gradual improvements in maternal and child health interventions" as possible explanation. However, alternative explanation—selection bias from large exclusion rate (76%)—not discussed. Were sicker/poorer women systematically excluded due to missing data? This could artificially lower LBW prevalence.

Discussion meets PLOS standards for interpretation, contextualization, and implications. However, could strengthen by addressing: Selection bias from 76% exclusion rate and impact on generalizability, Measurement error implications more explicitly, Regional heterogeneity and whether findings apply equally across all LMICs

**Accept with minor review**

This manuscript presents a rigorous, well-executed propensity score matching analysis addressing an important evidence gap in maternal and child health. The methodology is sound, findings are clearly presented, and implications for policy and practice are substantial. The work meets PLOS ONE standards for originality, technical rigor, and scientific importance.

Revisions addressing data discrepancies, clarifying matching procedures, and strengthening discussion of selection bias and measurement error will enhance the final publication. The study contributes meaningfully to understanding ANC's causal impact on birth outcomes and supports WHO recommendations for ANC8+ models across LMICs.
