# Supplementary material for: Estimating the effect of antenatal care models on birthweight in low- and middle-income countries: Evidence from a DHS-based propensity score matching analysis
Source: PLoS One. 2026 Aug 14;21(8):e0355685. doi: 10.1371/journal.pone.0355685 (PMC13475944; doi:10.1371/journal.pone.0355685)
Supplement: Peer review history

# Peer review: Decision Letter 0

**DOI:** 10.1371/journal.pone.0355685.r001

**Authors:** Sabita Tuladhar

21 Jun 2026

Please submit your revised manuscript by Aug 05 2026 11:59PM. If you will need more time than this to complete your revisions, please reply to this message or contact the journal office at plosone@plos.org. When you're ready to submit your revision, log on to https://www.editorialmanager.com/pone/ and select the 'Submissions Needing Revision' folder to locate your manuscript file.

- A letter that responds to each point raised by the academic editor and reviewer(s). You should upload this letter as a separate file labeled 'Response to Reviewers'.
- A marked-up copy of your manuscript that highlights changes made to the original version. You should upload this as a separate file labeled 'Revised Manuscript with Track Changes'.
- An unmarked version of your revised paper without tracked changes. You should upload this as a separate file labeled 'Manuscript'.

If you would like to make changes to your financial disclosure, please include your updated statement in your cover letter. Guidelines for resubmitting your figure files are available below the reviewer comments at the end of this letter.

If applicable, we recommend that you deposit your laboratory protocols in protocols.io to enhance the reproducibility of your results. Protocols.io assigns your protocol its own identifier (DOI) so that it can be cited independently in the future. For instructions see: https://journals.plos.org/plosone/s/submission-guidelines#loc-laboratory-protocols. Additionally, PLOS ONE offers an option for publishing peer-reviewed Lab Protocol articles, which describe protocols hosted on protocols.io. Read more information on sharing protocols at https://plos.org/protocols?utm_medium=editorial-email&utm_source=authorletters&utm_campaign=protocols.

As the corresponding author, your ORCID iD is verified in the submission system and will appear in the published article. PLOS supports the use of ORCID, and we encourage all coauthors to register for an ORCID iD and use it as well. Please encourage your coauthors to verify their ORCID iD within the submission system before final acceptance, as unverified ORCID iDs will not appear in the published article. *Only*  the individual author can complete the verification step; PLOS staff *cannot*  verify ORCID iDs on behalf of authors.

We look forward to receiving your revised manuscript.

Kind regards,

Sabita Tuladhar, PhD, MHealSc, MA

Academic Editor

PLOS One

Journal Requirements:

When submitting your revision, we need you to address these additional requirements.

1. Please ensure that your manuscript meets PLOS ONE's style requirements, including those for file naming. The PLOS ONE style templates can be found at

https://journals.plos.org/plosone/s/file?id=wjVg/PLOSOne_formatting_sample_main_body.pdf and

https://journals.plos.org/plosone/s/file?id=ba62/PLOSOne_formatting_sample_title_authors_affiliations.pdf

2. In the online submission form, you indicated that “The data for this study were obtained from the DHS program with a reasonable request. Thus, the one who needs the data supporting the findings of this study can get it in anonymised form from the DHS website at https://dhsprogram.com/Countries/ upon reasonable request in the same manner as the authors did”

All PLOS journals now require all data underlying the findings described in their manuscript to be freely available to other researchers, either 1. In a public repository, 2. Within the manuscript itself, or 3. Uploaded as supplementary information.

This policy applies to all data except where public deposition would breach compliance with the protocol approved by your research ethics board. If your data cannot be made publicly available for ethical or legal reasons (e.g., public availability would compromise patient privacy), please explain your reasons on resubmission and your exemption request will be escalated for approval.

3. If the reviewer comments include a recommendation to cite specific previously published works, please review and evaluate these publications to determine whether they are relevant and should be cited. There is no requirement to cite these works unless the editor has indicated otherwise.

4. Please review your reference list to ensure that it is complete and correct. If you have cited papers that have been retracted, please include the rationale for doing so in the manuscript text, or remove these references and replace them with relevant current references. Any changes to the reference list should be mentioned in the rebuttal letter that accompanies your revised manuscript. If you need to cite a retracted article, indicate the article’s retracted status in the References list and also include a citation and full reference for the retraction notice.

**Additional Editor Comments:**

**Methods – Study design, period, and population**

The study included women aged 15–49 years who had a live birth within five years preceding the survey. Pls confirm if the reference period was five, three or two years. It should be clarified whether all DHS surveys consistently measured the variables of interest across the 52 countries.

**Birthweight measurement**

Clarify how birthweights were obtained across DHS surveys:

- Were they measured using standardized weighing scales?
- Or based on maternal report (recall or health card)? Differences in measurement methods should be explicitly stated.

**Limitations**

Additional limitations should be acknowledged as applicable, including:

- Comparability of data across the 52 countries
- Variations in birthweight measurement approaches
- Potential recall bias in maternally reported birthweights

**Abbreviations**

Ensure abbreviations are defined at first use and applied consistently.

[Note: HTML markup is below. Please do not edit.]

Reviewers' comments:

Reviewer's Responses to Questions

**Comments to the Author**

1. Is the manuscript technically sound, and do the data support the conclusions?

Reviewer #1: Yes

Reviewer #2: Yes

**********

2. Has the statistical analysis been performed appropriately and rigorously?

Reviewer #1: Yes

Reviewer #2: Yes

**********

3. Have the authors made all data underlying the findings in their manuscript fully available?

The PLOS Data policy

Reviewer #1: Yes

Reviewer #2: Yes

**********

4. Is the manuscript presented in an intelligible fashion and written in standard English?

Reviewer #1: Yes

Reviewer #2: Yes

**********

Reviewer #1: Thank you for your nice work. I really enjoyed. The manuscript is clearly written. However, the authors need to adderess the following points:

(1) The discussion is superficial. The authors need high discussion.

(2) Implication for practice is missing.

(3) Recommondation part needs retuning

Reviewer #2: Introduction Major comment: Please elaborate about the burden of LBW in LMICs

Minor comment: Since COVID-19 is no more a public health concern, it might not be implacable to be included in the study.

Methods Preparing a flow diagram to explain sampling would be deme

How were the missing data for other than birthweight, ANC

records, and multiple births handled?

Other Information Please mention line number, which will make further review easier

**********

what does this mean?). If published, this will include your full peer review and any attached files.

If you choose “no”, your identity will remain anonymous but your review may still be made public.

**Do you want your identity to be public for this peer review?**  For information about this choice, including consent withdrawal, please see our Privacy Policy

Reviewer #1: No

Reviewer #2: **Yes:** Shristi Rijal

**********

[NOTE: If reviewer comments were submitted as an attachment file, they will be attached to this email and accessible via the submission site. Please log into your account, locate the manuscript record, and check for the action link "View Attachments". If this link does not appear, there are no attachment files.]

To ensure your figures meet our technical requirements, please review our figure guidelines: https://journals.plos.org/plosone/s/figures

You may also use PLOS’s free figure tool, NAAS, to help you prepare publication quality figures: https://journals.plos.org/plosone/s/figures#loc-tools-for-figure-preparation.

NAAS will assess whether your figures meet our technical requirements by comparing each figure against our figure specifications.

Attachment Submitted filename: Review_ANC_LBW.docx

# Author comment: Author response to Decision Letter 1

**DOI:** 10.1371/journal.pone.0355685.r002

25 Jun 2026

A point-by-point response to the editor and reviewer

Dear Dr Sabita Tuladhar, PhD, MHealSc, MA, Academic Editor

Thank you for your careful evaluation of our manuscript and for providing us with the opportunity to revise and resubmit our work. We are pleased to submit the revised version of our manuscript, entitled “Estimating the Effect of Antenatal Care Models on Birthweight in Low- and Middle-Income Countries: Evidence from a DHS-Based Propensity Score Matching analysis” (Manuscript ID: PONE-D-26-04747).

We sincerely appreciate the insightful and constructive comments provided by you and the reviewers. The feedback has been invaluable in strengthening the scientific rigor, methodological robustness, and overall presentation of the manuscript. In response, we have undertaken some revisions throughout the manuscript.

All comments have been addressed in a detailed point-by-point response document. For ease of evaluation, they have been highlighted throughout the revised manuscript using track changes, and the corresponding page and line numbers are provided in our responses to the reviewer’s comments.

We are grateful for the time and effort invested by the editor and a reviewer in improving our manuscript. We hope that the revisions adequately address all concerns and that the manuscript is now suitable for publication in PLOS ONE. We remain happy to consider any additional suggestions that may further strengthen the manuscript.

On the following pages, we provide our detailed responses to the reviewer’s comments.

With best regards,

Aklilu Habte Hailegebireal, MPH

Corresponding Author

akliluhabte57@gmail.com

Response to the additional editor’s comment

Comment: The study included women aged 15-49 years who had a live birth within five years preceding the survey. Pls confirm if the reference period was five, three or two years. It should be clarified whether all DHS surveys consistently measured the variables of interest across the 52 countries.

Response: Thank you for this important comment. We have clarified the reference period and the availability of study variables across the included DHS surveys. The analysis was restricted to women aged 15-49 years who had a live birth within the five years preceding each survey, consistent with the standard DHS birth recode dataset. We further verified that the key variables required for this study, including antenatal care utilization, birthweight, maternal sociodemographic characteristics, and other covariates, were available and measured using standardized DHS questionnaires across all 52 included countries. To ensure comparability, only surveys containing the variables necessary for defining antenatal care models, birthweight outcomes, and matching covariates were included in the analysis. We tried to make it clearer and highlighted in the method section of the ‘Revised manuscript with track changes’, Page 4, Line 122-128.

Comment 2: Clarify how birthweights were obtained across DHS surveys: Were they measured using standardized weighing scales? Or based on maternal report (recall or health card)? Differences in measurement methods should be explicitly stated.

Response: Thank you for this important observation. We have clarified the source and measurement of birthweight in the Methods section. In DHS surveys, birthweight is not measured directly by DHS interviewers. Instead, birthweight information is obtained either from the child’s health card or medical record when available, or from maternal recall when documentary records are unavailable. As measurement methods may vary across settings, we have acknowledged this as a potential source of measurement error and discussed it as a limitation of the study. Please make a look over the method section of the ‘Revised manuscript with track changes’, Page 7, Line 158-162.

Comment 3: Additional limitations should be acknowledged as applicable, including:

• Comparability of data across the 52 countries

• Variations in birthweight measurement approaches

• Potential recall bias in maternally reported birthweights

Response: Thank you for this valuable comment. We agree that these are important limitations that should be acknowledged. Accordingly, we have expanded the limitations section to discuss potential issues related to data comparability across the 52 included countries, variations in birthweight measurement approaches (health card/medical record versus maternal recall), and the possibility of recall bias in maternally reported birthweights. These additions have been incorporated and highlighted in the Strengths and limitations section of the ‘Revised manuscript with track changes’, Page 24, Lines 604-616.

Authors’ Response to Reviewer#1

General comment: Thank you for your nice work. I really enjoyed. The manuscript is clearly written. However, the authors need to address the following points:

Response: We sincerely appreciate your positive evaluation of our manuscript and your encouraging comments regarding its clarity and overall quality. We appreciate the time and effort invested in reviewing our work. We have carefully considered all comments and suggestions and have revised the manuscript accordingly. Detailed responses to each point are provided below, and all corresponding changes have been highlighted in the revised manuscript with track changes.

Comment 1: The discussion is superficial. The authors need a high discussion.

Response: Thank you for this constructive comment. Accordingly, we thoroughly revised and expanded the Discussion to provide a more comprehensive interpretation of the findings, compare our results with existing evidence from low- and middle-income countries and global literature, explore potential mechanisms underlying the observed associations, and discuss the public health and policy implications of adequate antenatal care utilization. We have also strengthened the discussion of the study's strengths and limitations. These revisions have substantially enhanced the depth and scholarly contribution of the Discussion section and are highlighted in the revised manuscript with track changes.

Comment 2: Implication for practice is missing.

Response: Thank you for this valuable observation. We agree that the implications for practice were not sufficiently explicit in the previous version of the manuscript. Accordingly, we have revised and expanded this section to clear out the practical and policy implications of our findings. Specifically, we highlighted the importance of strengthening access to and continuity of antenatal care services, promoting adherence to the WHO-recommended ANC8+ model, and prioritizing interventions that improve ANC utilization and quality of care in low- and middle-income countries. These revisions have been incorporated and highlighted in the Discussion section of the ‘revised manuscript with track changes’, Page 23, Line 577-591.

Comment 3: Recommendation part needs retuning

Response: Thank you for this constructive comment. We agree that the recommendation section requires further refinement to better reflect the study findings and their implications for policy and practice. Accordingly, we revised the recommendations to provide more specific, actionable, and evidence-informed guidance for policymakers, healthcare providers, and maternal health programs. The revised version is highlighted in the ‘conclusion’ section of the ‘revised manuscript with track changes’, Page 24, Line 618-626.

Thank you once again for your valuable time, thoughtful review, and important contributions to improving our manuscript.

_________________________________________________________________________________________________________END_______________________________________

THANK YOU!!!

Response to Reviewer #2 (Shristi Rijal) comment

Dear Shristi Rijal, we sincerely appreciate your positive evaluation of our manuscript and your encouraging comments regarding its clarity and overall quality. We appreciate the time and effort invested in reviewing our work.

Comment 1: Introduction Major comment: Please elaborate about the burden of LBW in LMICs

Response: Thank you for this valuable comment. We agree that the burden of low birthweight (LBW) in LMICs requires further elaboration. Accordingly, we expanded the Introduction to provide additional context on the magnitude of LBW in LMICs, its contribution to neonatal morbidity and mortality, adverse childhood growth and developmental outcomes, and its long-term health and socioeconomic consequences. These were highlighted in the ‘background’ section of the ‘revised manuscript with track changes’, Page 3, Line 68-78.

Comment 2: Preparing a flow diagram to explain sampling would be deme

Response: Thank you for this valuable suggestion. We agree that a flow diagram would improve the transparency of the sampling process and participant selection. Accordingly, we have developed and incorporated a detailed flow diagram (Figure 1) illustrating the sampling procedure, including the initial pooled DHS sample, application of eligibility criteria, exclusions, derivation of the final analytical sample, and classification of participants into the ANC4+ and ANC8+ exposure groups for propensity score matching analyses. The sampling procedure has been further elaborated in the “Sampling Techniques and Procedures” section of the ‘revised manuscript with track changes’ (Page 6, Lines 138-151).

Comment 3: How were the missing data for other than birthweight, ANC

records, and multiple births handled?

Response: Missing data were assessed for all study variables prior to analysis. Records with missing information on key variables, including birth weight, antenatal care (ANC) attendance, and plurality, were excluded because these variables were essential for defining the study population and conducting the planned analyses. For all other variables, the proportion of missing data was low (<2% in this scenario) and was therefore retained as missing. Analyses were conducted using complete-case observations for the variables included in each statistical model. No imputation procedures were performed, as the level of missingness was considered unlikely to affect the study findings.

We sincerely thank you for your thoughtful, constructive, and insightful comments provided on our manuscript. We greatly appreciate the time and effort invested in reviewing our work. All corresponding revisions have been incorporated and highlighted in the revised manuscript with track changes, with the relevant page and line numbers indicated for ease of reference.

_________________________________________________________________________________________________________END_______________________________________

THANK YOU!!!

Attachment Submitted filename: Response to reviewer.docx

# Peer review: Decision Letter 1

**DOI:** 10.1371/journal.pone.0355685.r003

**Authors:** Sabita Tuladhar

26 Jul 2026

Estimating the Effect of Antenatal Care Models on Birthweight in Low- and Middle-Income Countries: Evidence from a DHS-Based Propensity Score Matching analysis

PONE-D-26-04747R1

Dear Dr. Aklilu,

We’re pleased to inform you that your manuscript has been judged scientifically suitable for publication and will be formally accepted for publication once it meets all outstanding technical requirements.

Within one week, you’ll receive an e-mail detailing the required amendments. When these have been addressed, you’ll receive a formal acceptance letter and your manuscript will be scheduled for publication.

An invoice will be generated when your article is formally accepted. Please note, if your institution has a publishing partnership with PLOS and your article meets the relevant criteria, all or part of your publication costs will be covered. Please make sure your user information is up-to-date by logging into Editorial Manager at Editorial Manager® and clicking the ‘Update My Information' link at the top of the page. For questions related to billing, please contact billing support.

If your institution or institutions have a press office, please notify them about your upcoming paper to help maximize its impact. If they’ll be preparing press materials, please inform our press team as soon as possible -- no later than 48 hours after receiving the formal acceptance. Your manuscript will remain under strict press embargo until 2 pm Eastern Time on the date of publication. For more information, please contact onepress@plos.org.

Kind regards,

Sabita Tuladhar, PhD, MHealSc, MA

Academic Editor

PLOS One

**Comments to the Author**

Reviewer #2: All comments have been addressed

Reviewer #3: (No Response)

**********

2. Is the manuscript technically sound, and do the data support the conclusions?

Reviewer #2: Yes

Reviewer #3: Yes

**********

3. Has the statistical analysis been performed appropriately and rigorously?

Reviewer #2: Yes

Reviewer #3: Yes

**********

4. Have the authors made all data underlying the findings in their manuscript fully available?

The PLOS Data policy

Reviewer #2: Yes

Reviewer #3: Yes

**********

5. Is the manuscript presented in an intelligible fashion and written in standard English?

Reviewer #2: Yes

Reviewer #3: Yes

**********

Reviewer #2: Great work on the revision and also liked the flow diagram. Also, the author addressed other comments on the background and has further worked on the methodology section.

Reviewer #3: Thank you for the opportunity to review the revised version of this manuscript, entitled “Estimating the Effect of Antenatal Care Models on Birthweight in Low- and Middle-Income Countries: Evidence from a DHS-Based Propensity Score Matching Analysis.”

The authors have substantially improved the manuscript and have addressed the major concerns raised during the previous round of review. The study addresses an important public health question and uses a large, multi-country DHS dataset and propensity score matching methods to examine the relationship between antenatal care models and birthweight outcomes. Overall, the methodology is appropriate, the analyses are generally rigorous, and the conclusions are broadly supported by the findings. The manuscript is also clearly presented and has potential relevance for maternal and child health policy and practice.

I recommend publication after minor revision, which are attached in the above documents. These are relatively minor issues that can be addressed through clarification, consistency checks, and strengthening the limitations and interpretation sections. Overall, the manuscript is methodologically sound and makes a meaningful contribution to the evidence on antenatal care models and birth outcomes in low- and middle-income countries. I look forward to seeing the revised version.

**********

what does this mean?). If published, this will include your full peer review and any attached files.

If you choose “no”, your identity will remain anonymous but your review may still be made public.

**Do you want your identity to be public for this peer review?**  For information about this choice, including consent withdrawal, please see our Privacy Policy

Reviewer #2: **Yes:** Shristi Rijal

Reviewer #3: No

**********

Attachment Submitted filename: Reviewer Feedback Estimating the Effect of ANC Models.docx

# Editor report: Acceptance letter

**DOI:** 10.1371/journal.pone.0355685.r004

**Authors:** Sabita Tuladhar

PONE-D-26-04747R1

PLOS One

Dear Dr. Hailegebireal,

I'm pleased to inform you that your manuscript has been deemed suitable for publication in PLOS One. Congratulations! Your manuscript is now being handed over to our production team.

At this stage, our production department will prepare your paper for publication. This includes ensuring the following:

* All references, tables, and figures are properly cited

* All relevant supporting information is included in the manuscript submission,

* There are no issues that prevent the paper from being properly typeset

You will receive further instructions from the production team, including instructions on how to review your proof when it is ready. Please keep in mind that we are working through a large volume of accepted articles, so please give us a few days to review your paper and let you know the next and final steps.

Lastly, if your institution or institutions have a press office, please let them know about your upcoming paper now to help maximize its impact. If they'll be preparing press materials, please inform our press team within the next 48 hours. Your manuscript will remain under strict press embargo until 2 pm Eastern Time on the date of publication. For more information, please contact onepress@plos.org.

You will receive an invoice from PLOS for your publication fee after your manuscript has reached the completed accept phase. If you receive an email requesting payment before acceptance or for any other service, this may be a phishing scheme. Learn how to identify phishing emails and protect your accounts at https://explore.plos.org/phishing.

If we can help with anything else, please email us at customercare@plos.org.

Thank you for submitting your work to PLOS ONE and supporting open access.

Kind regards,

PLOS ONE Editorial Office Staff

on behalf of

Dr. Sabita Tuladhar

Academic Editor

PLOS One
